# The Clinical Picture and Fecundity of Primary and Recurrent Ovarian Endometriosis with Family History: A Retrospective Analysis

**DOI:** 10.3390/jcm12051758

**Published:** 2023-02-22

**Authors:** Bingning Xu, Li Lin, Yongchao Pan, Pei Chen, Chaoshuang Ye, Li Zhao, Yang Jin, Yong Zhou, Ruijin Wu

**Affiliations:** Department of Gynecology, Women’s Hospital, School of Medicine, Zhejiang University, Hangzhou 310006, China

**Keywords:** endometrioma, familial anamnesis, fertility, recurrence, reproductive performance

## Abstract

This study aims to evaluate the role of endometriosis family history on the clinical manifestation and fertility performance of primary and recurrent endometriosis. In total, 312 primary and 323 recurrent endometrioma patients with a histological diagnosis were included in this study. Family history was significantly correlated with recurrent endometriosis (adjusted OR: 3.52, 95% CI: 1.09–9.46, *p* = 0.008). Patients with a family history showed a significantly higher proportion of recurrent endometriosis (75.76% vs. 49.50%), higher rASRM scores, higher incidence of severe dysmenorrhea, and severe pelvic pain than the sporadic cases. Recurrent endometrioma showed statistical increase in rASRM scores, percentage of rASRM Stage IV, dysmenorrhea, dyschezia, those undergoing semi-radical surgery or unilateral oophorosalpingectomy, postoperative medical treatment, e with a positive family history, while a decrease in the incidence of asymptomatic phenomena and those undergoing ovarian cystectomy compared to those with primary endometriosis. The naturally conceived pregnancy rate was higher in primary endometriosis compared to recurrent endometriosis. Compared to recurrent endometriosis with a negative family history, recurrent endometriosis with a positive family history had a higher incidence of severe dysmenorrhea, chronic pelvic pain, a higher spontaneous abortion rate, and a lower natural pregnancy rate. Primary endometriosis with a family history presented a higher incidence of severe dysmenorrhea than those without a family history. In conclusion, endometriosis patients with a positive family history presented a higher pain severity and lower conception probability compared to the sporadic cases. Recurrent endometriosis showed further-exacerbated clinical manifestations, more pronounced familial tendency, and lower pregnancy rates than primary endometriosis.

## 1. Introduction

Endometriosis is a chronic, benign gynecological disorder affecting 10–15% of reproductive-age women worldwide. It is usually associated with chronic pelvic pain, dysmenorrhea, dyspareunia, dyschezia, and infertility [1]. Although endometriosis remains an enigmatic disease, there is mounting evidence that genetic and epigenetic conditions are conducive to the development of endometriosis. Genome wide association studies (GWAS) on populations of the United States, Australia, Japan, and Europe have identified susceptibility loci and potential candidate genes [2,3,4].

There is consistent evidence that a positive family history of endometriosis is a significant risk factor, with first-degree relatives of affected patients at a four- to ten-fold increased risk of developing the disease [5], and with studies of twins illustrating heritability may be as high as almost 50% [6]. In addition, higher recurrence rates following surgery were also found in women with a positive familial anamnesis for endometriosis [7].

Ovarian endometriosis is probably the most common type, estimated to affect up to 44% of endometriosis patients [8]. It is suggested that laparoscopic conservative surgery is the gold standard treatment for ovarian endometrioma [9,10]. However, surgical excision is associated with a considerable burden of relapse. The recurrent rate of endometriosis following surgery may be as high as 40–50% for 5 years [11]. Recurrent endometriosis substantially impaired the quality of life (QoL) of women and imposed healthcare costs.

To date, few studies investigated family history’s influence on the clinical presentation of primary and recurrent endometrioma. This study aimed to gain further insight into the correlation between family history and clinical manifestations and the fertility of primary and recurrent endometriosis.

## 2. Materials and Methods

### 2.1. Ethics Statement

This study was approved by the Ethics Committee of Women’s Hospital, School of Medicine, Zhejiang University (IRB-20190021-R). Written informed consent was obtained from all participants.

### 2.2. Patient Population

From January 1, 2007 to 31 December 2018, patients undergoing primary or recurrent ovarian endometriosis surgery in our hospital were included in this study. Surgical modalities included conservative, semi-radical surgery (defined as lesion excision plus hysterectomy with preservation of bilateral or unilateral ovary in this article) and unilateral oophorosalpingectomy via laparotomy or laparoscopy.

Inclusion criteria were: ovarian endometrioma having been confirmed surgically and histologically. All enrolled patients did not receive steroid hormone medication at least 6 months prior to surgery.

Recurrent endometriosis was clinically suspected when patients met one of the following criteria: had positive signs (pelvic mass/pelvic nodulations at pelvic examination) relating to the pelvic area that reappeared or worsened at the preoperative level after postsurgical remission, ultrasound diagnosis of recurrence, serum CA125 re-increased following postoperative decrease, with or without clinical symptoms (dysmenorrhea, dyspareunia, or dyschezia) recurring or even being aggravated after a postoperative remission period of at least 3 month [12,13,14]. The final diagnosis would be confirmed via pathology.

Exclusion criteria were: pathology report informed malignant transformation, adenomyosis, endometriotic cyst rupture, hypertension, diabetes mellitus, infection diseases, autoimmune disease, tumor, serious internal disease, abnormal uterine bleeding.

### 2.3. Methods

**Figure 1:**
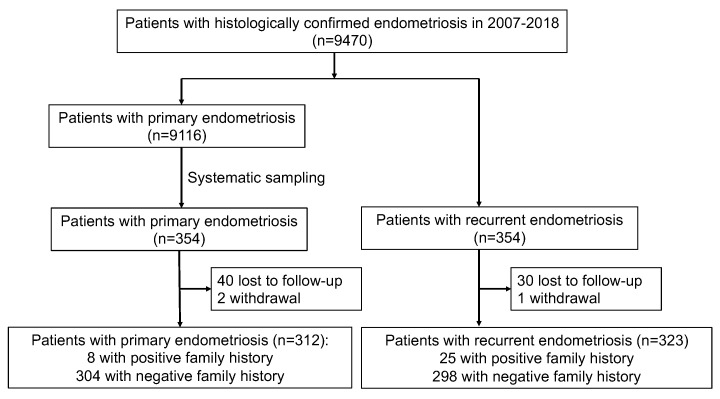
Flowchart of patients’ enrollment.

We identified 9470 histologically confirmed endometrioma women; 354 patients experienced ovarian endometriosis relapse and underwent repeat surgery, and 354 out of 9116 women undergoing primary endometriosis were selected via a systematic sampling method (Figure 1). Systematic sampling method: since 9116 could not be divisible by 354, 266 subjects were removed via a simple random sampling method, and the remaining 8850 patients were randomly numbered and evenly allocated to 354 groups. Every group randomly selected one patient, then 354 individuals were systematically sampled as the primary endometriosis group.

Patients were approached via telephone to complete the self-developed questionnaire by a trained interviewer, who asked for information including fertility condition (mode of conception, pregnancy outcome, number of pregnancies, etc.) after surgery, endometriosis in all first- (i.e., mother, sisters, and daughters) and second-degree relatives (maternal and paternal aunts).

Information about daughters over the age of 17 was included because endometriosis is known to be hormonally influenced [15]. Grandmothers and nieces were excluded since few of the grandmothers could provide an accurate medical history and nieces had more distant relationships compared with other second-degree relatives. Information about relatives of the included subjects was confirmed by themselves via a telephone interview.

The following data were recorded: age (years), body mass index (BMI, kg/m^2^), preoperative serum CA125 level, education status, family history, recurrence interval, symptoms, surgical modality, postoperative medical treatment, disease stage according to the revised American Society for Reproductive Medicine (rASRM) classification and scores, and pregnancy outcomes.

The visual analogue scale (VAS) 0 to 10 point was used to measure pain symptoms (0 = absent, 10 = unbearable; a VAS score of 7 or more was taken to indicate that a symptom was ‘severe’). Pregnancy outcomes were included as follows: successful pregnancy, spontaneous abortion, premature birth, live birth, and ongoing pregnancy.

Pregnancy of the subjects was diagnosed via ultrasound examination; the following conditions were excluded in the fertility evaluation: hysterectomy, bilateral salpingectomy, salpingemphraxis, and male factor infertility.

### 2.4. Statistical Analysis

Quantitative data were expressed as a mean and standard deviation (SD). Qualitative data were expressed as a frequency and percentage. We used the Student’s *t*-test or the Wilcoxon–Mann–Whitney test for continuous variables and the chi-squared or Fisher’s exact test for categorical variables. Multivariable logistic regression was used to study the association between endometrioma recurrence and family history. Factors known to influence endometriosis recurrence were considered as potential confounders, such as dysmenorrhea, mode of surgery, postoperative pregnancy, and postoperative medical treatment [12,13,16]. The unadjusted odds ratio (crude odds ratio, cOR) and the adjusted odds ratio (aOR) with a 95% confidence interval (95% CI) were calculated. Univariable logistic regression models were used to assess the correlation between first- and second-degree relatives and endometriosis recurrence. The odds ratio (OR) was used to express the strength of association, together with a 95% CI. The statistical analyses were performed on the software SPSS Statistics 25.0. Values were considered statistically significant if *p* < 0.05.

## 3. Results

Compared to primary endometrioma, the recurrent endometriosis individuals had higher rASRM scores and percentages of rASRM Stage IV. The proportion of asymptomatic patients in primary and recurrent endometrioma was 23.72% vs. 4.64% (*p* < 0.001), respectively. Patients who underwent a second surgery had a significantly higher incidence of pain symptoms such as dysmenorrhea and dyschezia and a higher percentage of patients taking postoperative medical treatment. As for the modality of surgery, the percentage of ovarian cystectomy was higher in primary endometrioma (*p* < 0.001), while semi-radical surgery or unilateral oophorosalpingectomy was higher in recurrent endometrioma (*p* < 0.001). The number of postoperative pregnancy cases in the primary and recurrent endometriosis groups was 138 and 179, with successful pregnancy rates of 71.74% (99/138) and 55.87% (100/179), respectively. (Table 1).

A positive family history correlated with a higher rate of endometriosis recurrence. After adjusting for potential confounding factors, patients with a positive family history were at least three times more likely to have recurring endometriosis than sporadic patients (crude OR =4.309, 95%CI:1.456–12.363; adjusted OR = 3.520, 95% CI: 1.089–9.457, *p* = 0.008).

Patients with familial anamnesis have higher rASRM scores than those without a family history (87.45 ± 30.98 vs. 54.53 ± 33.11). Severe pain symptoms such as severe dysmenorrhea (36.36% vs. 14.62%) and severe pelvic pain (27.27% vs. 12.13%) were more prevalent in the positive family history group than in the negative family history group. The proportion of recurrent endometriosis was higher in the endometriosis with a positive family history group (75.76%) than in those with a negative family history (49.50%). (Table 2)

Although the percentage of affected first- and second-degree relatives was higher in recurrent endometriosis than in primary endometriosis, the difference was not statistically significant. The incidence of endometriosis increased with kinship in both recurrent and primary endometriosis groups. (Table 3) The incidence of endometrioma in first-degree relatives of recurrent endometriosis was significantly higher than in second-degree relatives (OR = 2.8, 95% CI [1.1, 7.0], *p* = 0.026). In primary endometriosis, there was no difference in the incidence of endometrioma between first- and second-degree relatives (OR = 1.4, 95% CI [0.3, 5.7], *p* = 0.68).

In the primary endometriosis group, patients with a positive family history have a significantly higher incidence of severe dysmenorrhea than those with a negative family history (37.5% vs. 12.83%, *p* = 0.044). (Table 4).

In the recurrent endometriosis group, women with familial anamnesis had a higher prevalence of severe dysmenorrhea (36% vs. 16.44%, *p* = 0.01) and severe chronic pelvic pain (28% vs. 11.41%, *p* = 0.01) than women without familial anamnesis. Albeit non-significantly, the recurrent endometriosis patients with a positive family history had a shorter recurrence interval (4.64 ± 3.36 years) than those without a family history (6.10 ± 3.99 years) (Table 5).

There were 138 primary endometriosis patients and 179 recurrent endometriosis patients who wanted to conceive naturally following conservative endometriosis surgery. The pregnancy rate was calculated as the ratio of the number of patients with successful pregnancy to the number of patients attempting to conceive naturally (99/138 and 100/179 for primary and recurrent endometriosis, respectively). The abortion rate was the ratio of spontaneous pregnancy losses to the number of gravidities. Similarly, the preterm birth rate, term birth rate, and ongoing pregnancy rate were calculated in the same way. The number of gravidities for primary and recurrent endometriosis patients was 100 and 123, respectively.

The natural pregnancy rate was obviously higher in the primary endometriosis group compared to the recurrent group (71.74% vs. 55.87%, *p* = 0.04) (Table 6).

The number of gravidities for recurrent endometriosis women with and without a family history was 11 and 112, respectively. Recurrent endometriosis women with a positive familial anamnesis presented a higher spontaneous abortion rate (27.27% vs. 8.04%, *p* = 0.04) and lower pregnancy rate (36.36% vs. 58.60%, *p* = 0.04) compared to patients without a positive family history (Table 7).

In primary endometriosis patients, there was only one case with a positive family history that was conceived naturally and delivered at term. Of the 137 sporadic primary endometriosis patients, 98 patients had a successful pregnancy, 8 underwent a spontaneous abortion, 10 had a preterm birth, 65 had a full-term birth, and 16 were an ongoing pregnancy. There was no significant difference between the two groups.

Forty-five patients underwent assisted reproductive technology (ART), among which twelve were primary endometriosis patients and thirty-three were recurrent patients. The cumulative live birth rate (CLBR) is defined as the chance of pregnancy resulting in a live birth after each ART cycle. The rate of miscarriage (16.7% vs. 15.2%) and CLBR (41.7% vs. 39.4%) showed no statistical difference between primary and recurrent endometriosis.

No fetal abnormalities cases were observed in this study cohort, which was probably due to low incidence or patients’ request for confidentiality.

## 4. Discussion

Endometriosis is a polygenic/multifactorial disease showing heritable tendencies, and genetic predisposition is considered to be an important contributor to the pathogenesis [17,18]. According to the prediction of the polygenic model [17], the greater the severity of a polygenic disorder, the greater the underlying genetic liability. Thus, an increase in the proportion of affected relatives would increase the probability of severe endometriosis among probands. In our study, patients with positive family history proved more likely to have severe pain symptoms, such as severe dysmenorrhea and severe chronic pelvic pain, irrespective of primary or recurrent endometriosis.

Accumulating studies were concerned about the familial tendency of endometriosis. A recent nationwide study quantified a 2.75-fold elevated familial risk of endometriosis among patients with and without affected siblings. The familial risk increased with the degree of genetic relatedness, and the highest risk was with affected twins [19]. In the present study, the incidence of endometriosis decreasing with lower kinship levels in both primary and recurrent endometriosis suggested that endometriosis follows a polygenic inheritance pathway [18]. Moreover, recurrent endometriosis presented more notable familial aggregation than primary endometriosis, with evidence of a higher percentage of familial cases (7.74% vs. 2.56%) and a higher incidence of endometrioma in first-degree relatives than second-degree relatives of recurrent endometriosis women (3.73% vs. 1.13%, *p* = 0.026). Several studies reported that the prevalence of endometriosis in first-degree relatives of patients was 5.9–9.4% [20,21]. In our study, the familial risk for endometriosis in first-degree relatives of primary and recurrent endometriosis was 1.6% and 3.7%, respectively, lower than those reported in the literature. We speculated that the reason may lie in the different methods of patient selection. All ovarian endometriosis patients enrolled in the present study were pathologically diagnosed; pelvic pain and infertility were their main reasons for hospital presentation. First-degree relatives of endometriosis women enrolled in our study were all surgically diagnosed. Moreover, the average age of primary and recurrent endometriosis women was 31 and 33 in our data, respectively, and they had less likelihood of having daughters with a clinical diagnosis of endometriosis, which could be another reason for decreasing the sample size of first-degree relatives with endometriosis.

Postoperative recurrence of endometriosis remains a serious challenge. Recent studies suggested that patients with ovarian endometrioma were more likely to have the same subtype at relapse, with more severe manifestations in a substantial proportion of patients. The recurrence interval was independent of lesion subtype at the initial surgery [22,23]. Consistently, recurrent endometrioma patients in our study presented a more severe manifestation compared with primary cases, for example, they manifested as higher rASRM scores and showed a higher proportion of severe endometriosis (rASRM Stage IV), which resulted in a higher percentage of postoperative medical treatment (55.73% vs. 43.91%) and more likelihood of greater surgical coverage (semi-radical surgery or unilateral oophorosalpingectomy). One year post-surgery in our study, patients with rASRM Stage III-IV endometriosis were normally advised to take postoperative medical treatment for prevention recurrence if they did not want pregnancy. The tendency for recurrent endometriosis to present with more severe symptoms suggested that the disease may progress overtime, whether or not the lesion was removed [22].

Endometriosis can adversely affect fecundity via different mechanisms, and up to 50% of women with endometriosis have fertility problems [24]. Several studies suggested that endometrioma did not cause infertility per se, while ovarian surgery might negatively affect ovarian reserves, which would lead to infertility [24]. In the present study, the spontaneous pregnancy rate of recurrent endometrioma (55.87%) was lower than primary endometrioma (71.74%), which may be due to the greater damage to the ovarian reserve in secondary surgery than in primary surgery [25]; surgery associated pelvic inflammation may be another reason. Moreover, our study demonstrated that familial cases showed a lower spontaneous pregnancy rate (36.36% vs. 58.60%) and higher abortion rate (27.27% vs. 8.04%) than sporadic cases in the recurrence group. We speculated that the lower natural pregnancy rate in familial cases of recurrent endometrioma might be related to more severe manifestations, such as severe pelvic pain, which could lead to the sexual dysfunction of patients. The relationship between fertility performance and family history needs further investigation.

It is recommended that patients who have no immediate desire for pregnancy should consider medical treatment to reduce endometriosis-associated pain [13]. For women who attempt to or decide to conceive, the ART technique is generally considered when surgical and pharmacological treatments are ineffective. Notably, the optimal time for ART after endometriosis surgery is within 2 years [26]. The effect of endometriosis on ART outcomes remains controversial [27]. Reducing the number of available embryos for transfer, the surgical history of endometriosis may or not be associated with lower pregnancy rates [28,29]. As for the recurrence of endometriosis, Vercellini et al. suggested that the pregnancy rate was reduced after repetitive surgery; the results of IVF were not far behind those undergoing reoperation [30]. In the present study, the CLBR after ART was higher in women with primary endometriosis than those with recurrent endometriosis, albeit non-significantly.

It seems to be established beyond reasonable doubt that the longer the follow-up, the higher the rate of endometriosis recurrence. In most studies, the duration of follow-up ranged from 2 years up to 5 years, but few studies were longer than 5 years [31]. Our data suggested that the recurrence interval was 4.64 ± 3.36 years in familial cases and 6.10 ± 3.99 years in sporadic cases. Therefore, health-care professionals should provide endometriosis patients with continuous postoperative follow-up support and be aware of the symptoms and time points that suggest the need for surgical intervention, especially for those sporadic cases with higher recurrent risks.

The main limitation of the current study is that it is a single-center study, so that the sample size of certain subgroups was limited; future large-sample, multicenter research is required to validate our results. Another limitation is that third-degree relatives of patients with endometriosis were not included, because most of the grandmothers could not provide the medication history precisely and nieces had more distant relationships compared with other second-degree relatives.

In conclusion, endometriosis women with a positive family history had a significantly higher likelihood of reporting a higher pain severity and reduced pregnancy rate compared to the sporadic cases. Moreover, the recurrent endometriosis showed a higher percentage of severe clinical manifestation, more pronounced familial tendency, and lower spontaneous pregnancy rates than primary endometriosis. It is urgent to increase our attention toward endometriosis patients with a positive family and to provide them with individualized clinical treatment, pregnancy counselling, and postoperative follow-up programs.

## Figures and Tables

**Table 1:** Characteristics of primary and recurrent endometriosis patients.

	Primary Endometriosis (*n* = 312)	Recurrent Endometriosis (*n* = 323)	*p*-Value
Age (y)	31.29 ± 5.261	33.55 ± 5.730	NS
College degree or higher	227	234	NS
BMI (kg/m^2^)	21.5 ± 3.85	21.59 ± 3.04	NS
CA125 (IU/L)	93.66 ± 73.829	88.55 ± 10.43	NS
rASRM scores	48.41 ± 28.37	67.17 ± 33.80	0.003
rASRM classification			<0.001
Stage II	25 (8.01%)	3(0.93%)	
Stage III	142 (45.51%)	68 (21.05%)	
Stage IV	145 (46.47%)	252 (78.02%)	
Dysmenorrhea	217 (69.55%)	251 (77.71%)	0.02
Severe dysmenorrhea (VAS ≥ 7)	42 (13.46%)	58 (17.96%)	NS
Chronic pelvic pain	57 (18.27%)	71 (21.98%)	NS
Severe chronic pelvic pain (VAS ≥ 7)	41 (13.14%)	41 (12.69%)	NS
Dyspareunia	32 (10.26%)	44 (13.62%)	NS
Dyschezia	7 (2.24%)	17 (5.26%)	0.04
Asymptomatic	74 (23.72%)	15 (4.64%)	<0.001
Ovarian cystectomy	280 (89.74%)	235 (72.76%)	<0.001
Semi-radical surgery or unilateral oophorosalpingectomy	32 (10.26%)	88 (27.25%)	<0.001
Postoperative medical treatment	137 (43.91%)	180(55.73%)	0.003
Positive family history	8 (2.56%)	25(7.74%)	0.003
Postoperative pregnancy	99 (71.74%)	100 (55.87%)	0.04

*p* < 0.05 means statistically significant. NS means not significant.

**Table 2:** Characteristics of patients with positive and negative family history.

	Patients with Positive Family History (*n* = 33)	Patients without Positive Family History (*n* = 602)	*p*-Value
Age (y)	32.62 ± 6.20	31.94 ± 5.22	NS
College degree or higher	26 (78.78%)	435 (72.26%)	NS
BMI (kg/m^2^)	22.41 ± 4.15	22.10 ± 4.02	NS
CA125 (IU/L)	63.77 ± 1	88.97 ± 10.19	NS
rASRM cores	87.45 ± 30.98	54.53 ± 33.11	0.02
rASRM classification			NS
stage II	1 (3.03%)	27 (4.49%)	
stage III	9 (27.27%)	201 (33.39%)	
stage IV	23 (69.70%)	374 (62.13%)	
Dysmenorrhea	25 (75.75%)	443 (73.59%)	NS
Severe dysmenorrhea (VAS ≥ 7)	12 (36.36%)	88 (14.62%)	0.01
Chronic pelvic pain	11 (33.33%)	117 (19.46%)	NS
Severe chronic pelvic pain (VAS ≥ 7)	9 (27.27%)	73 (12.13%)	0.01
Dyspareunia	2 (9.09%)	73 (12.13%)	NS
Dyschezia	2 (6.06%)	22 (3.65%)	NS
Asymptomatic	0	89 (14.78%)	0.01
Ovarian cystectomy	28 (84.84%)	487 (80.90%)	NS
Semi-radical surgery or unilateral oophorosalpingectomy	5 (15.15%)	115 (19.10%)	NS
Postoperative medical treatment	13 (39.39%)	304 (50.50%)	NS
Recurrent endometriosis	25 (75.76%)	298 (49.50%)	0.003

*p* < 0.05 means statistically significant. NS means not significant.

**Table 3:** Incidence of endometriosis in first- and second-degree relatives of recurrent and primary endometriosis.

	Recurrent Endometriosis (*n* = 323)	Primary Endometriosis (*n* = 312)	Odds Ratio [95% CI]	*p*-Value
First-degree relatives				
Mothers	10/323 (3.1%)	3/312 (1.0%)	3.3 [0.9, 21.1]	NS
Sisters	8/165 (4.9%)	2/158 (1.2%)	4.0 [0.8, 19.0]	NS
Daughters	1/21 (4.8%)	0/11 (0%)	/	NS
Total	19/509 (3.7%)	5/481 (1.6%)	3.7 [1.4, 10.0]	NS
Second-degree relatives				
Maternal aunts	4/275 (1.45%)	2/205 (0.97%)	1.5 [0.3, 8.3]	NS
Paternal aunts	2/257 (0.78%)	1/184 (0.54%)	1.4 [0.1, 15.9]	NS
Total	6/532 (1.13%)	3/389 (0.84%)	1.5 [0.4, 5.9]	NS

NS means not significant.

**Table 4:** Comparison of characteristics between primary endometriosis patients with and without family history.

	Patients with Positive Family History (*n* = 8)	Patients without Positive Family History (*n* = 304)	*p*-Value
Age (y)	32.01 ± 5.68	31.27 ± 5.25	NS
College degree or higher	6 (75%)	221 (72.70%)	NS
BMI (kg/m^2^)	22.64 ± 4.44	21.47 ± 3.83	NS
CA125 (IU/L)	92.42 ± 84.76	93.69 ± 73.31	NS
rASRM cores	50.55 ± 29.58	48.35 ± 28.34	NS
rASRM classification			NS
Stage II	1 (12.5%)	24 (7.90%)	
Stage III	3 (37.5%)	139 (45.72%)	
Stage IV	4 (50%)	141 (46.38%)	
Dysmenorrhea	6 (75%)	211 (69.41%)	NS
Severe dysmenorrhea (VAS ≥ 7)	3 (37.5%)	39 (12.83%)	0.04
Chronic pelvic pain	2 (25%)	55 (18.09%)	NS
Severe chronic pelvic pain (VAS ≥ 7)	2 (25%)	39 (12.83%)	NS
Dyspareunia	1 (12.5%)	31 (10.20%)	NS
Dyschezia	0	7 (2.30%)	NS
Asymptomatic	0	74 (24.34%)	NS
Ovarian cystectomy	7 (87.5%)	273 (89.80%)	NS
Semi-radical surgery or unilateral oophorosalpingectomy	1 (12.5%)	31 (10.20%)	NS
Postoperative medical treatment	3 (37.5%)	134 (44.08%)	NS

*p* < 0.05 means statistically significant. NS means not significant.

**Table 5:** Comparison of characteristics between recurrent endometriosis patients with and without family history.

	Patients with Positive Family History (*n* = 25)	Patients without Positive Family History (*n* = 298)	*p*-Value
Age (y)	34.00 ± 6.08	33.51 ± 5.7	NS
College degree or higher	20 (80%)	214 (71.81%)	NS
Recurrence interval (y)	4.64 ± 3.36	6.10 ± 3.99	NS
BMI (kg/m^2^)	22.43 ± 3.26	21.52 ± 3.02	NS
CA125 (IU/L)	83.54 ± 12.91	88.97 ± 10.19	NS
rASRM cores	87.45 ± 38.49	65.47 ± 33.38	NS
rASRM classification			NS
stage II	0	3 (1.0%)	
stage III	6 (24.0%)	62 (20.81%)	
stage IV	19 (76%)	233 (78.19%)	
Dysmenorrhea	19 (76.0%)	232 (77.85%)	NS
Severe dysmenorrhea (VAS≥ 7)	9 (36%)	49 (16.44%)	0.01
Chronic pelvic pain	9 (36%)	62 (20.81%)	NS
Severe chronic pelvic pain (VAS≥ 7)	7 (28%)	34 (11.41%)	0.01
Dyspareunia	2 (8%)	42 (14.09%)	NS
Dyschezia	2 (8%)	15 (5.03%)	NS
Asymptomatic	0	15 (5.03%)	NS
Ovarian cystectomy	21 (84%)	214 (71.81%)	NS
Semi-radical surgery or unilateral oophorosalpingectomy	4 (16%)	84 (28.19%)	NS
Postoperative medical treatment	10 (40%)	170 (57.05%)	NS

*p* < 0.05 means statistically significant. NS means not significant.

**Table 6:** Comparison of naturally conceived pregnancy outcomes between primary and recurrent endometriosis patients.

	Primary Endometriosis (*n* = 138)	Recurrent Endometriosis (*n* = 179)	*p*-Value
Successful pregnancy	99 (71.74%)	100 (55.87%)	0.04
Spontaneous abortion	8 (8%)	12 (9.76%)	NS
Premature birth	10 (10%)	14 (11.4%)	NS
Live birth	66 (66%)	86 (69.9%)	NS
Ongoing pregnancy	16 (16%)	11 (8.9%)	NS

*p* < 0.05 means statistically significant. NS means not significant.

**Table 7:** Comparison of naturally conceived pregnancy outcomes between recurrent endometriosis patients with and without family history.

	Patients with Positive Family History (*n* = 22)	Patients without Positive Family History (*n* = 157)	*p*-Value
Successful pregnancy	8 (36.36%)	92 (58.60%)	0.04
Spontaneous abortion	3 (27.27%)	9 (8.04%)	0.04
Premature birth	1 (9.09%)	13 (11.61%)	NS
Live birth	6 (54.55%)	80 (71.43%)	NS
Ongoing pregnancy	1 (9.09%)	10 (8.93%)	NS

*p* < 0.05 means statistically significant. NS means not significant.

## Data Availability

Data were obtained from the Women’s Hospital, School of Medicine, Zhejiang University. According to relevant regulations, the data could not be shared but can be requested from the corresponding author.
